# Smooth constrained block matching algorithm for the evaluation of diaphragm deformation in speckle-tracking ultrasound

**DOI:** 10.3389/fmed.2026.1842503

**Published:** 2026-05-29

**Authors:** Qiang Li, Peiming Zhang, Xiong Ye, Qiaohong Liu

**Affiliations:** 1 Henan Center for Drug Evaluation and Inspection, Zhengzhou, Henan, China; 2 School of Health Science and Engineering, University of Shanghai for Science and Technology, Shanghai, China; 3 School of Clinical Medicine, Shanghai University of Medicine & Health Sciences, Shanghai, China; 4 School of Medical Instruments, Shanghai University of Medicine & Health Sciences, Shanghai, China

**Keywords:** diaphragm, displacement estimation, speckle tracking algorithm, strain calculation, ultrasound strain imaging

## Abstract

**Background:**

This study presents a novel ultrasound speckle-tracking algorithm as a technical feasibility demonstration for sub-pixel diaphragm deformation analysis.

**Methods:**

Six healthy beagles were enrolled in this study. Images were captured by a portable ultrasound system from the right and left hemidiaphragms. SCBM-based algorithms were designed to obtain the inter-frame/cumulative horizontal and vertical displacements, as well as the global strain of the diaphragm. The algorithm combined the inter-frame integer displacement estimation of the SCBM method, the sub-pixel level displacement estimation of the optical flow method, the cumulative displacement calculation of the continuous tracking algorithm, and the sub-pixel displacement accumulation correction algorithm, which is called SOCS. Global diaphragm strain was calculated from the displacements estimated by SOCS. Displacement and strain estimates were compared between the right and left hemidiaphragms.

**Results:**

SOCS algorithm can track the contraction and relaxation of the diaphragm by following the respiratory movement continuously. No significant difference was found between the right and left diaphragms when the mean or median cumulative displacement of all points in the ROI region of interest was used to approximate the diaphragmatic displacement (*p* > 0.05). Global diaphragm strain differed significantly between the left and right hemidiaphragms (all *p* < 0.05).

**Conclusion:**

This study presents a pilot feasibility demonstration of an ultrasound speckle-tracking method for diaphragm deformation analysis. The proposed method enabled estimation of inter-frame and cumulative displacement in the horizontal and vertical directions, as well as approximate global strain on two imaging planes. In this small cohort, left diaphragmatic strain differed significantly from right diaphragmatic strain. These findings suggest that diaphragm strain estimates may be sensitive to imaging-plane selection.

## 1 Introduction

The diaphragm is a musculotendinous structure that forms a dome-shaped boundary between the thoracic and abdominal cavities and accounts for 60–80% of respiratory effort. In addition to respiratory functions, diaphragm also participates in various physiological activities such as vomiting, childbirth, and excretion. Conditions such as central nervous system disorders, lesions above the C7 spinal cord segment, chronic obstructive pulmonary disease, and asthma can cause diaphragm dysfunction (1–4), resulting in sleep deprivation, breathing difficulties, and respiratory failure may occur. Therefore, effective evaluation of diaphragmatic function is clinically important.

However, despite advances in B-mode and M-mode ultrasound for diaphragm assessment, a quantitative, reproducible, and operator-independent metric for diaphragm strain at sub-pixel resolution remains lacking, which limits broader clinical adoption. As a real-time, non-invasive, and convenient diagnostic method, ultrasound can directly evaluate diaphragmatic movement function (5–7). There have been many related studies (8, 9) based on B-mode and M-mode ultrasound in recent years. But B-mode ultrasound can only reflect the two-dimensional structural information of the diaphragm and cannot provide the mechanical characteristics of the diaphragm (10). M-mode ultrasound can only show the one-dimensional motion information of the diaphragm on the ultrasound beam and cannot capture the two-dimensional motion information of the diaphragm (11). Furthermore, B- and M-mode ultrasound have obvious angle-of-incidence dependence and require the operator to have extensive experience.

Ultrasound speckle-tracking imaging technology obtains tissue mechanical information, e.g., displacement and strain, to determine tissue lesions and the degree of lesion, which has been successfully applied to breast (12), thyroid (13), heart (14), intravascular plaque (15), and other parts. In 2013, Ye et al. (16) proposed the concept of diaphragmatic deformation analysis. Hatam et al. (17) evaluated diaphragmatic strain using commercial myocardial tracking software. Mark et al. (18) pointed out that diaphragmatic strain imaging may become a new method for evaluating diaphragmatic function in the future. Ye et al. (19) proposed a normalized cross-correlation (NCC) algorithm for diaphragm deformation. However, NCC-based block matching is known to be vulnerable to speckle decorrelation in highly deforming regions and is constrained to integer-pixel displacement resolution, which limits the precision of strain estimation. Moreover, prior approaches did not explicitly incorporate the spatial inertia of soft-tissue motion as a regularization prior, leaving room for improvement in tracking robustness.

**Figure 1:**
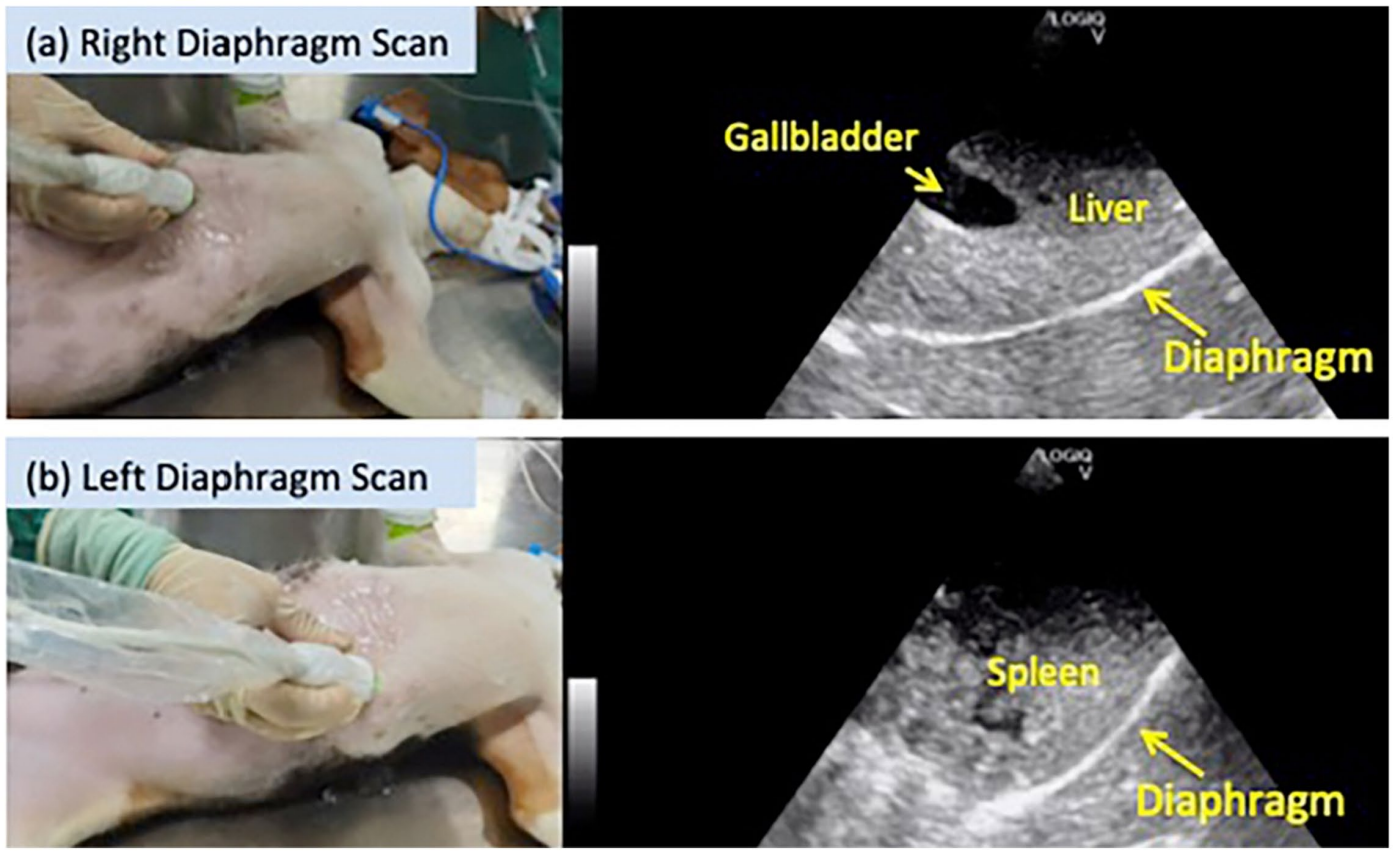
Ultrasound sections of the right and left diaphragm obtained with a portable ultrasound machine from beagle. **(a)** Right diaphragm of beagle; **(b)** Left diaphragm of beagle.

In this study, as a preliminary feasibility study, we propose a novel speckle-tracking algorithm (termed SOCS) that integrates smooth-constraint block matching, optical-flow sub-pixel estimation, continuous tracking, and a sub-pixel cumulative-displacement correction step. To our knowledge, this combination has not been previously applied to diaphragm deformation analysis. The algorithm is evaluated for sub-pixel-level displacement and global strain estimation in six healthy beagles (Figure 1).

## 2 Materials and methods

Six healthy beagles aged 3–6 months were enrolled in this study. All animal procedures were reviewed and approved by the Institutional Animal Care and Use Committee of the Shanghai University of Medicine & Health Sciences (protocol number: 2022-GZR-01-330621197407185776). Anesthesia was induced with intramuscular atropine (0.1 mg/kg) and Zoletil (7–25 mg/kg). After endotracheal intubation, anesthesia was maintained with intravenous xylazine (0.5–1 mg/kg) as needed. Mechanical ventilation was set to synchronized intermittent mandatory ventilation (SIMV) mode with a respiratory rate of 10 breaths/min, tidal volume of 200–300 mL, plateau pressure of 10 cmH₂O, and positive end-expiratory pressure of 5 cmH₂O. Right and left hemidiaphragmatic dynamic movements were acquired using a 3–5 MHz convex-array probe (LOGIQ V2, General Electric Healthcare, Horton, Norway) at a frame rate of 30 frames per second. For each animal and each imaging plane, three consecutive respiratory cycles were recorded, and each cycle contained approximately 50–60 frames. A total of 12 DICOM cine sequences (6 animals × 2 sections) were saved for subsequent offline speckle-tracking analysis.

### 2.1 Basic process of tracking diaphragm deformation

**Figure 2:**
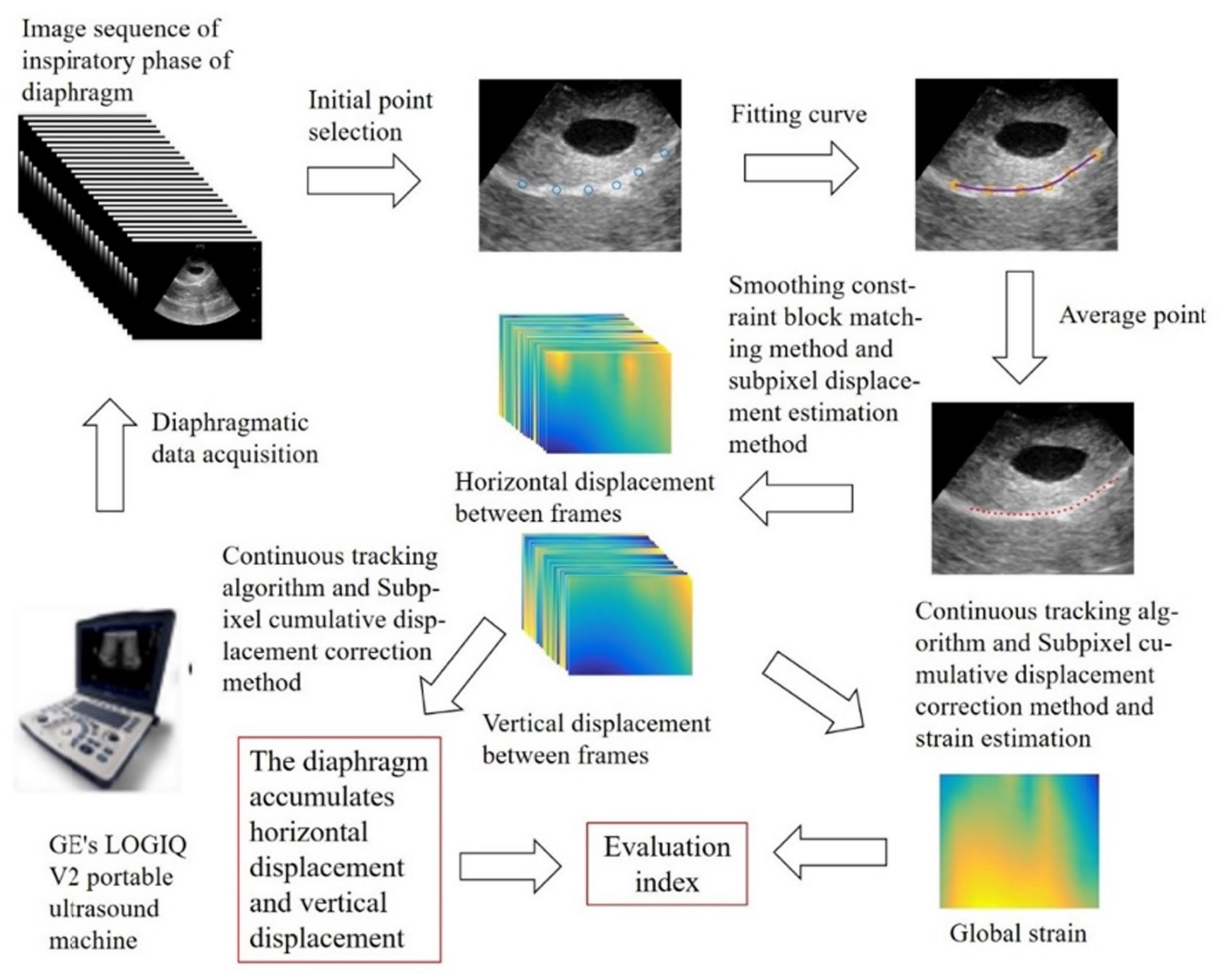
Workflow of diaphragm speckle-tracking analysis, including ROI initialization, curve fitting, block tracking, cumulative displacement estimation, and global strain calculation.

The basic process of diaphragm deformation is shown in Figure 2:

- Collect a series of data on the beagle diaphragm using the LOGIQ V2 portable ul-trasound system. Extract the inspiratory phase sequence image manually;
- Six initial points were manually selected by an experienced operator (>5 years of experience). The points were placed along the strongly echogenic diaphragmatic line from the lateral insertion to the central tendon;
- Twenty-four equally spaced points were automatically selected along the fitted curve as the centers of the tracking blocks;
- Use the proposed speckle-tracking algorithm to track the 24 matching blocks;
- Calculate the overall inter-frame and cumulative displacement of the diaphragm in the horizontal and vertical directions;
- Calculate the global strain of the diaphragm.

### 2.2 Diaphragm deformation tracking algorithm

#### 2.2.1 Integer displacement estimation

**Figure 3:**
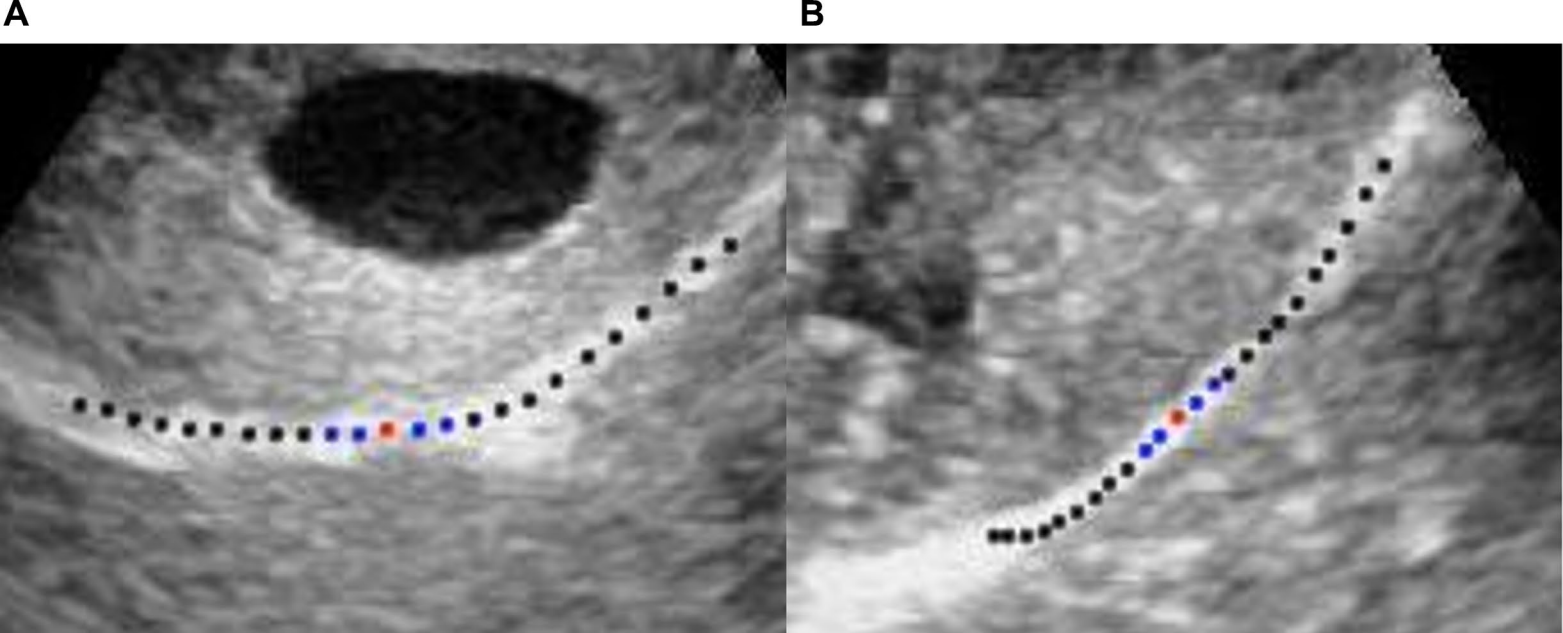
Illustration of the smoothness constraint used in local neighborhood motion estimation: **(a)** Right diaphragm and **(b)** left diaphragm.

The smooth constrained block matching method is proposed to estimate the integer displacement between the frames of the diaphragm. Generally, the speckle-tracking algorithm relies on the stability of the ultrasound spots. When the spots move to a highly decorrelated region, the related motion information will be lost, resulting in inaccurate motion estimation during speckle tracking. Moreover, soft tissue motion has a certain spatial inertia. Prior knowledge of soft tissue motion physiology is used to address the decorrelation problem of the spots (20, 21). Since the soft tissue motion or stress field is continuous, smooth constraints can be applied to local motion estimation and target motion can be constrained by local motion (22, 23). As shown in Figure 3, the black dots represent all the points to be matched in the ROI, the red dot represents the current point to be tracked, and the blue dots represent the surrounding neighborhood points. Therefore, the speckle tracking problem can be transformed into a minimal optimization problem that combines a block matching method (24) and the smooth constraints, resulting in the regularized variational model in Equation 1, as follows.

$$\begin{array}{l} H(x,\hat{d}(x))=\sum_{x\in \Omega }\mathrm{SAD}(x,\hat{d}(x))\\ +\beta \sum_{c_{l}\in C_{l}}{\left\|\hat{d}(x_{i})-\hat{d}(x_{j})\right\|}^{2} \end{array}$$ (1)

Where:

-
x is a pixel position in the image. The notation $\hat{d}(x)$ represents the estimated displacement vector at position x .
-
Ω is the set of all pixel positions (target points) within the diaphragm ROI.
-
$\mathrm{SAD}(x,\hat{d}(x))$ is the sum of absolute differences between the block centered at x in the reference frame and the block centered at $x+\hat{d}(x)$ in the target frame.
-
$c_{l}$ denotes a pair of neighboring positions $\left(x_{i},x_{j}\right)$ that belong to the set $C_{l}$ , where $C_{l}$ is the set of all such neighboring pairs within Ω (using 8-neighborhood). The index l runs over all pairs.
-
$\hat{d}(x_{i})$ and $\hat{d}(x_{j})$ are the displacement vectors estimated at the two neighboring positions $x_{i}$ and $x_{j}$ .
-
∥·∥ is the Euclidean ( $L_{2}$ ) norm.
-
β is a positive regularization parameter (set to β=0.01 in this study) that controls the strength of the smoothness constraint.

The first term measures block matching similarity, and the second term penalizes large differences between displacements of neighboring pixels, enforcing spatial smoothness. Using the smoothness term in this formulation is justified by the continuity of soft-tissue deformation: within the small spatial scale of an 8-neighborhood (approximately 3–5 mm), the local displacement field can be approximated as locally affine. Therefore, penalizing large differences between neighboring displacements suppresses outliers caused by speckle decorrelation while retaining true motion information. The total cost $H(x,\hat{d}(x))$ is minimized over $\hat{d}(x)$ to obtain the optimal displacement field.

### 2.3 Subpixel displacement estimation

#### 2.3.1 Optical flow method for subpixel displacement estimation

Optical flow (25) is an important method for analyzing image motion which can express the changes in an image. The optical flow method can achieve sub-pixel accuracy directly for speckle tracking, but matching errors may occur when the ultrasound image has too much noise. To address this issue, a smooth-constraint block matching method combined with the optical flow is proposed for diaphragm ROI tracking, as follows.

**Figure 4:**
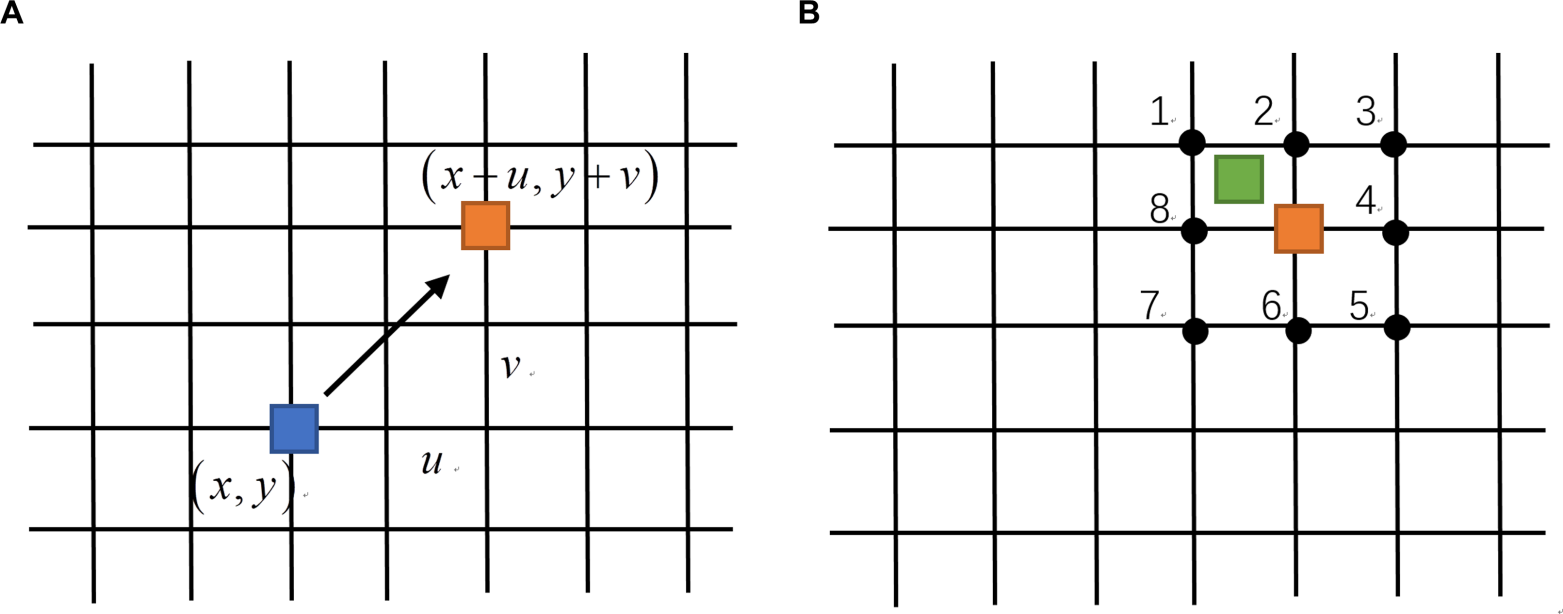
Schematic illustration of optical-flow-based sub-pixel displacement estimation after integer displacement initialization. **(a)** Block matching method. **(b)** Optical flow method.

First, the smooth constraint block matching method is used to calculate the diaphragm ROI inter-frame integer displacement, as shown in Figure 4a. Assuming the initial point (blue block) is at the position (x,y) , its horizontal and vertical displacements are u and v, respectively. Then, the optical flow is used to calculate sub-pixel displacement. The initial point is no longer at the blue block position but at the orange block position (x+u,y+v) . The optical flow method solves the sub-pixel displacement not on the next frame’s entire image but within the square enclosed by black points 1 (x+u−1,y+v+1) , points 2 (x+u,y+v+1) , points 3 (x+u+1,y+v+1) , points 4 (x+u+1,y+v) , points 5 (x+u+1,y+v−1) , points 6 (x+u,y+v−1) , points 7 (x+u−1,y+v−1) , and points 8 (x+u−1,y+v) , as shown in Figure 4b. The green block in Figure 4b represents the estimated motion position information.

#### 2.3.2 Continuous tracking algorithm

**Figure 5:**
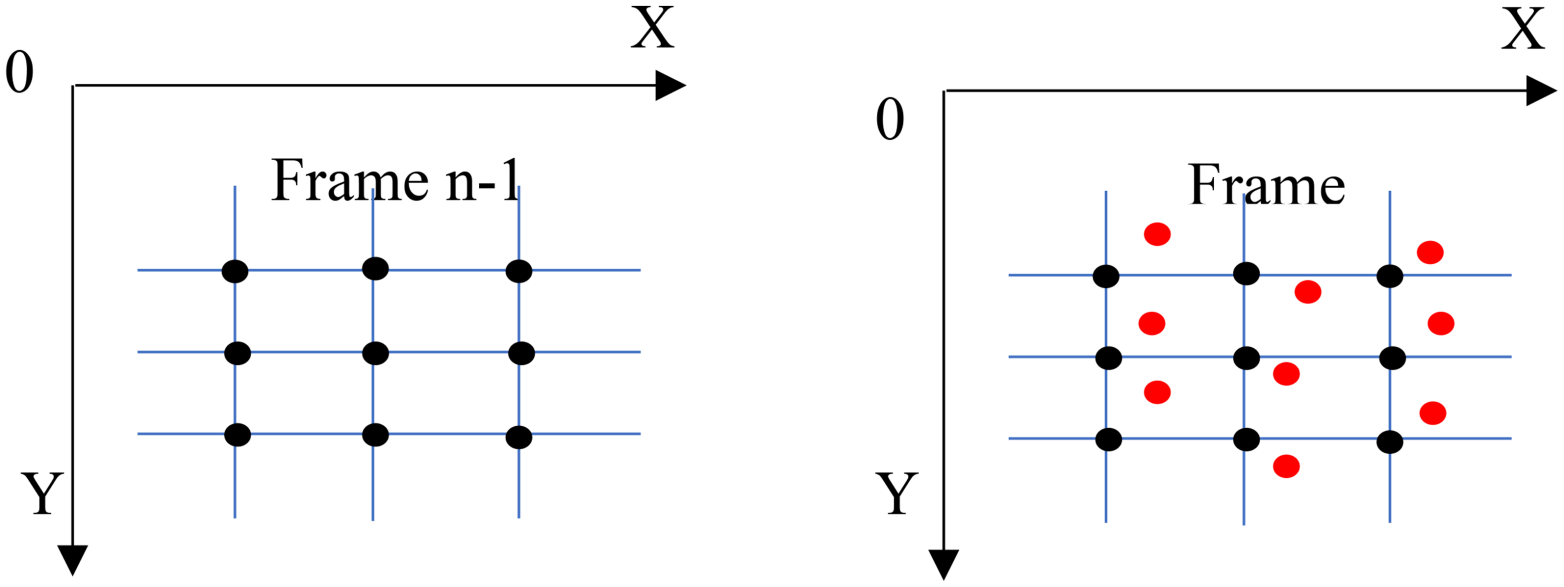
Diagram of the continuous tracking algorithm.

In order to obtain cumulative displacement of all blocks to be matched in diaphragm ROI under different Windows, continuous tracking algorithm (19) should be used to continuously track the same diaphragm region. As shown in Figure 5, specific description as follows:

- The interframe displacement grid point to be calculated is represented by a black dot and the coordinate of the dot is assumed to be (x,y) ;
- The interframe displacement (horizontal displacement and vertical displacement) of the dot in n-1 frame and n frame, and in n frame and n + 1 frame are represented by u1 , v1 and u2 , v2 respectively by block matching method;
- Since the diaphragm is in motion all the time, the black dot selected in frame n-1 has moved to a new position in frame n, which is represented by the red dot and its coordinates are assumed to be $\left(x_{1},y_{1}\right)$ , Among them: $x_{1}=x+u1,y_{1}=y+v1$ ;
- The horizontal and vertical displacements of the red dots were calculated using block matching method and represented by $\;u2^{\prime }$ , $v2^{\prime }$ ;
- Finally, the actual horizontal displacement and vertical displacement between frames at frame n and n + 1 are $\;u2^{\prime }$ , $v2^{\prime }$ , instead of u2 , v2 , and the cumulative horizontal displacement and vertical displacement of diaphragm at frame n-1 to n + 1 are $\;u1+u2^{\prime }$ , $v1+v2^{\prime }$ , respectively.

#### 2.3.3 Sub-pixel displacement cumulative correction algorithm

Since the input image is a two-dimensional matrix and the tracking algorithm can only handle pixel information corresponding to integer positions, the sub-pixel displacement of a frame would be automatically rounded to integers leading to cumulative errors. Therefore, a sub-pixel cumulative displacement correction method is proposed to overcome this problem. The algorithm is described as follows.

- Initialize uu and vv to 0;
- Point p with coordinates (x,y) is selected as the center of the initial block to be tracked when the inter-frame is calculated, where xandy are positive integers. The horizontal and vertical displacements from frame N−1 to N are represented by U1 and V1 , respectively;
-
U1 and V1 which are estimated at frame N−1 are rounded to $U1^{\prime }$ and $V1^{\prime }$ . Let $u1=U1-U1^{\prime }$ , $v1=V1-V1^{\prime }$ and update uu=uu+u1 , vv=vv+v1;
- If uu and vv are greater than 1, then uu=uu−1 , vv=vv−1 , U1=U1+1 , V1=V1+1 . If uu and vv are less than 1, then uu=uu+1 , vv=vv+1 , U1=U1−1 , V1=V1−1 . Else uu , vv , U1 , and V1 remain unchanged;
- Point p is updated by x=x+U1 , y=y+V1 ;
- Starting from step 2, repeat the same process for frame N until the last frame.

### 2.4 Global diaphragm strain calculation

To obtain the global strain of the diaphragm during inspiration, the distance between all adjacent points within the ROI is calculated by the above displacement calculation method and summed to obtain L_0_. Then, the speckle-tracking algorithm is used to track to the last frame of the sequence, and the distance between adjacent points is calculated again and summed to obtain L_t_. L_0_ and L_t_ can be approximated as the diaphragm length at the beginning of inhalation and the end of exhalation, respectively. The global strain (GS) of the diaphragm can be calculated by the following formula.

$$\mathrm{GS}=\frac{L_{t}-L_{0}}{L_{0}}$$

**Figure 6:**
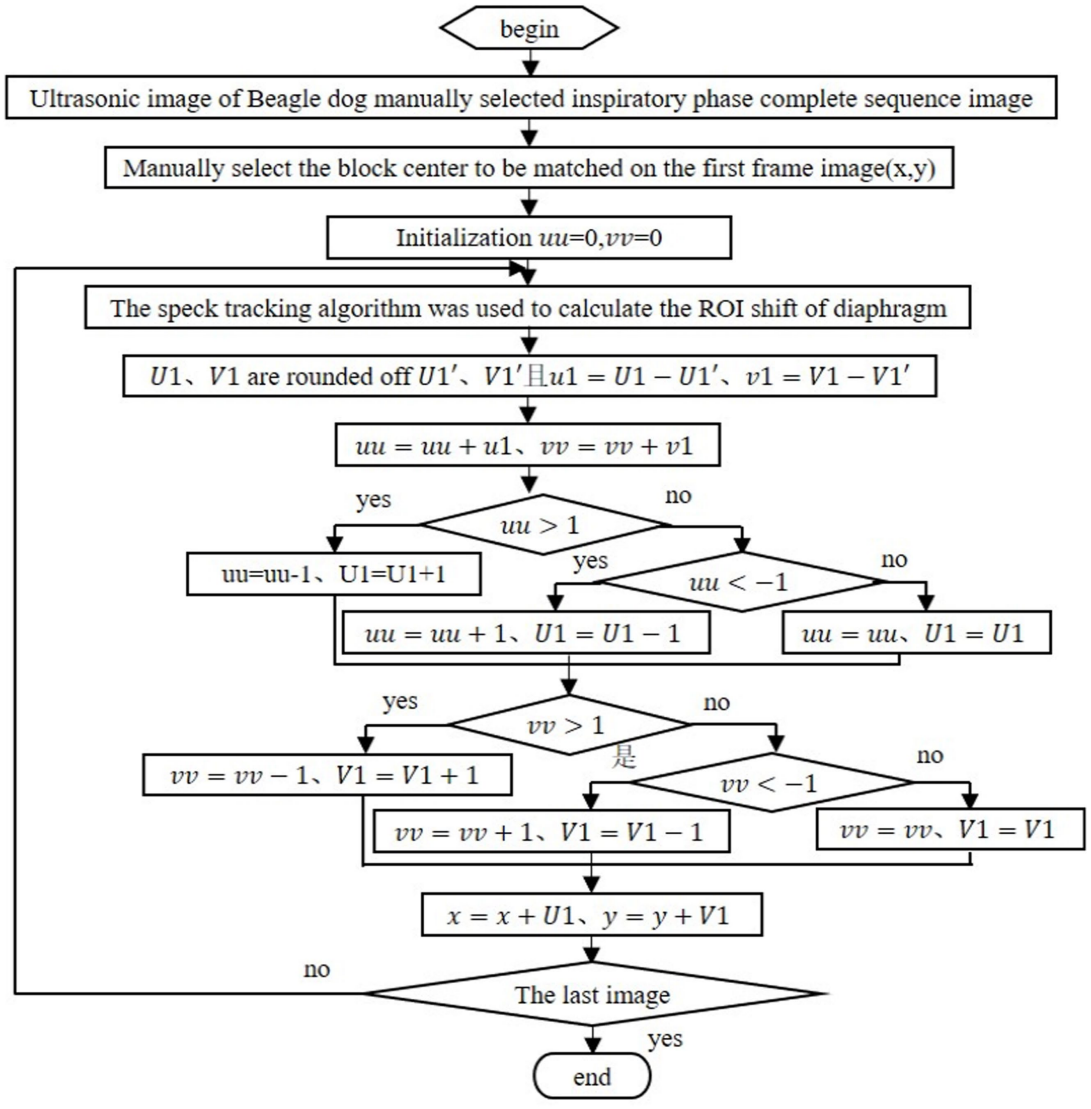
Flow chart of the sub-pixel cumulative-displacement correction algorithm.

The calculated strain is an approximate longitudinal global strain based on a polyline discretization of the curved diaphragm contour. This discretization is justified because the diaphragm appears as a curved echogenic line in the B-mode image, and its primary deformation during respiration is longitudinal stretching/shortening along this contour. Transverse and shear components are not captured by this simplified model (Figure 6).

### 2.5 Implementation details and statistical analysis

All experiments were performed on a Windows 10 workstation with an Intel Core i5-5200U CPU at 2.7 GHz and 4 GB RAM. The algorithms were implemented in MATLAB R2016a. All algorithm parameters were fixed prior to analysis: matching block size = 35 × 35 pixels; search window = ±7 pixels in both horizontal and vertical directions; smoothness weight (*β* in Equation 1) = 0.01. Sub-pixel displacement estimation used a pyramidal Lucas–Kanade optical flow method with three pyramid levels and a 5 × 5 integration window. Convergence was defined as a displacement update smaller than 0.01 pixel or a maximum of 30 iterations. The source code is available from the corresponding author upon reasonable request (Table 1).

**Table 1:** Per-animal global diaphragm strain values estimated using the SOCS algorithm.

Algorithm type	Sample	Right	Left
SOCS	1	−0.2204489	−0.1118820
	2	−0.3490045	−0.1956612
	3	−0.2210899	−0.0869266
	4	−0.1936362	−0.0835438
	5	−0.1796482	−0.1269940
	6	−0.1709477	−0.0749203

Statistical analysis was performed using SPSS 26.0 (IBM Corporation, NY, USA). Normality of the data was assessed using the Shapiro–Wilk test. For normally distributed data, a paired t-test was used for within-group comparisons; for non-normally distributed data, the Wilcoxon signed-rank test was applied. No correction for multiple comparisons was applied because of the pilot/feasibility nature of this study (all comparisons are considered exploratory). All tests were two-sided, and a *p*-value < 0.05 was considered statistically significant.

## 3 Results

**Figure 7:**
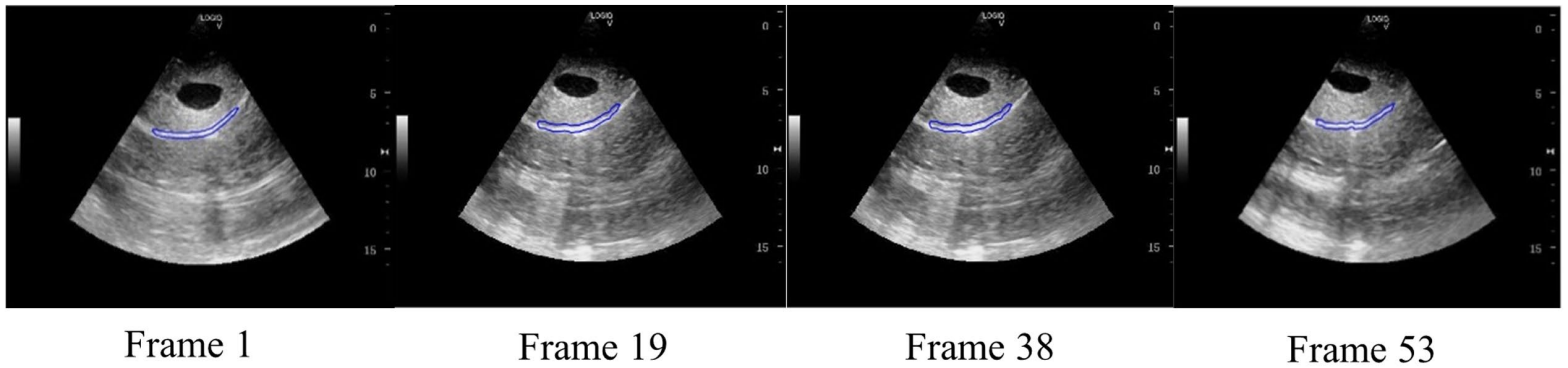
Continuous tracking of the diaphragm ROI during an inspiratory phase.

Figure 7 shows the results of continuous tracking of the diaphragm ROI during one respiratory cycle using the speckle-tracking algorithm based on smooth-constrained block matching. The diaphragm ROI at frame 1 was manually selected. The ROI at frame 53 was shorter than that at frame 1, indicating that the diaphragm was in a contracted state during inspiration.

**Figure 8:**
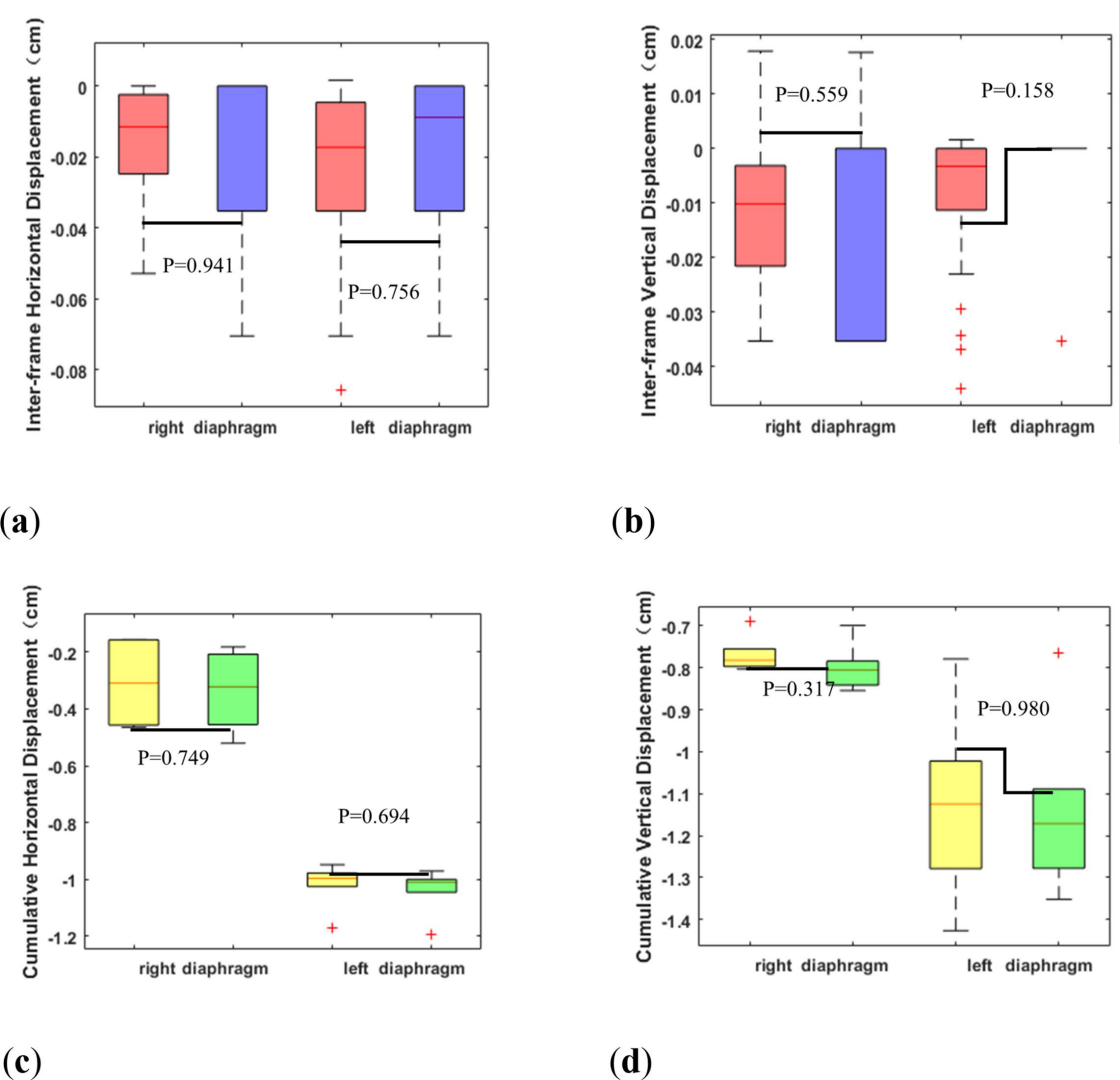
Boxplots of inter-frame and cumulative diaphragm displacement estimated by the SOCS algorithm, summarized using the mean or median of all tracked ROI points for the right and left hemidiaphragms. **(a)** Inter‑frame horizontal displacement values of the right and left diaphragm; **(b)** Inter‑frame vertical displacement between the right and left diaphragm; **(c)** Cumulative horizontal displacement between the right and left diaphragm; **(d)** Cumulative vertical displacement of the right and left diaphragm.

Figure 8 summarizes the inter-frame and cumulative horizontal and vertical displacement measures for the right and left hemidiaphragms, using the mean or median of all tracked ROI points to approximate diaphragm displacement. No significant difference was found between the right and left hemidiaphragms for these displacement-based summary measures (*p* > 0.05).

**Figure 9:**
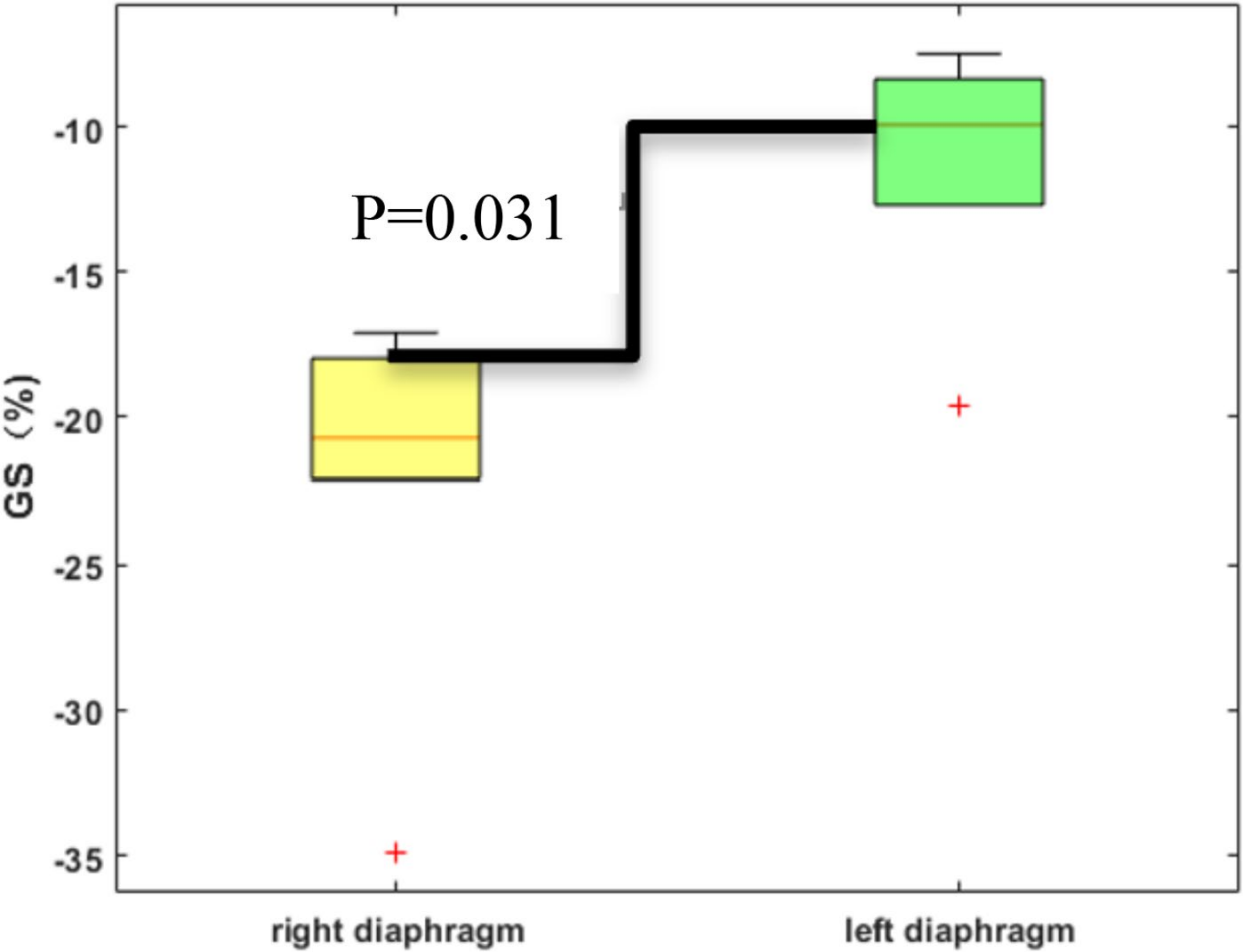
Comparison of global diaphragm strain between the right and left hemidiaphragms.

The global strain values for the right and left hemidiaphragms are summarized in Table 2. The right diaphragm showed a median strain of −0.207 (IQR: −0.220 to −0.180), while the left diaphragm showed a median strain of −0.099 (IQR: −0.127 to −0.084). A Wilcoxon signed-rank test revealed a significant difference between the two sides (median difference = −0.109, 95% CI: −0.153 to −0.053, *p* = 0.031). Figure 9 illustrates the distribution of global strain values in the right and left hemidiaphragms and is consistent with the significant between-side difference reported above.

**Table 2:** Summary statistics for global diaphragm strain in the right and left hemidiaphragms estimated using the SOCS algorithm (*n* = 6 per side).

Side	Median (IQR) strain	95% CI of median difference (right – left)	*p*-value
Right	−0.207 (−0.220, −0.180)	(−0.153, −0.053)	0.031
Left	−0.099 (−0.127, −0.084)		

The median difference (right – left) was −0.109, estimated using the Hodges–Lehmann method.

## 4 Discussion

The diaphragm is an important respiratory muscle in the human body, and various clinical conditions can lead to diaphragm dysfunction, which can directly endanger life. Therefore, it is necessary to develop a method for evaluating diaphragm function, and ultrasound is widely used for this purpose due to its non-invasiveness and convenience. Nowadays, diaphragm strain imaging can evaluate diaphragm function under different physiological or pathological conditions which is superior to the traditional B-mode and M-mode ultrasound. Therefore, in this study, a speckle tracking algorithm based on diaphragm strain imaging are developed for diaphragm function evaluation. The SOCS algorithm combined the inter-frame integer displacement estimation of the SCBM method, the sub-pixel level displacement estimation of the optical flow method, the cumulative displacement calculation of the continuous tracking algorithm, and the sub-pixel displacement accumulation correction algorithm. It should be emphasized that the primary goal of this study is to demonstrate the technical feasibility of the SOCS algorithm rather than to establish clinically validated reference values.

By using the proposed algorithm, the inter-frame or cumulative displacement and global strain are estimated. Some difference analyses were conducted, and the following observations were obtained. The algorithms can successfully track diaphragm ROI motion, The diaphragmatic interframe displacement and cumulative displacement can be approximated by the mean or median of the interframe and cumulative displacement at all points in ROI (that is, the *p* value is greater than 0.05). For diaphragm global strain, significant differences in strain between the two sections are observed when estimating diaphragm strain values using SOCS algorithm (*p* < 0.05). This indicates that there is a significant difference between the left and right diaphragms in the assessment of diaphragmatic function by using diaphragmatic strain.

The observed difference between left and right hemidiaphragmatic strain warrants careful interpretation. On the one hand, anatomical and physiological asymmetries — including the position of the liver under the right hemidiaphragm, asymmetric muscle-fiber orientation, and differential phrenic-nerve innervation — may produce genuine biomechanical differences between the two sides, consistent with prior reports of side-related variations in diaphragmatic structural and biomechanical properties (26, 27). On the other hand, this difference could partly reflect technical factors such as differences in imaging-plane orientation, ROI placement, and tracking errors arising from depth-dependent image quality. Given the pilot nature of this study, we cannot definitively separate physiological from technical contributions. Future validation against an independent reference standard (e.g., fluoroscopy or tagged MRI) is needed to clarify the origin of this observed difference.

Several limitations of this study should be acknowledged. First, the primary objective was to demonstrate technical feasibility, not clinical translation. Therefore, the study has significant shortcomings in clinical applicability, validation, and generalizability. Second, the sample size of n = 6 healthy beagles is small and was determined based on prior feasibility studies. This limits statistical power and the generalizability of results to clinical or pathological populations. The present study was designed as a pilot feasibility study, not a definitive clinical trial. Third, the smoothness prior assumes local affinity, which may bias strain estimates at regions with steep gradients (e.g., diaphragmatic insertions). Fourth, inter-operator variability and repeated measurements were not performed in this study; manual point selection was conducted by a single experienced operator. Future studies should include independent observers and repeated ROI initialization to assess reproducibility. Additionally, the global strain calculation discretizes the diaphragmatic contour into piecewise-linear segments and reports only longitudinal strain; transverse and shear components are ignored. This approximation is acceptable for a pilot feasibility study but may underestimate complex deformations. The study only included healthy beagles; therefore, the expected performance in pathological conditions (e.g., diaphragm dysfunction, mechanical ventilation) and sensitivity to reduced motion amplitude or abnormal strain patterns remain unknown. Translational studies in patient cohorts are needed. Quantitative performance metrics such as tracking error (RMSE), drift reduction, runtime, and sensitivity to noise or speckle decorrelation were not assessed in this pilot study due to the lack of a ground-truth reference and dedicated noise simulation. Tracking failure rate and confidence intervals for displacement estimation were not systematically assessed. This is a limitation of the current study. An ablation study to separate the contributions of the smooth-constraint block matching, optical-flow sub-pixel estimation, and cumulative-displacement correction was not performed. Therefore, the relative importance of each component remains unknown. Future work should include a systematic ablation analysis using synthetic or phantom data with known ground truth.

Recent ultrasound-based diaphragm studies using real-world data have provided complementary information. Dag et al. (26) used shear-wave elastography (SWE) to assess diaphragm thickness and stiffness in dialysis patients. Sinanoğlu et al. (27, 28) evaluated diaphragm properties in malnourished children and those with nocturnal enuresis. These studies characterize static tissue properties at a single respiratory phase. Our SOCS method offers a complementary advantage: dynamic in-plane kinematics (displacement and global strain) at sub-pixel resolution over the entire respiratory cycle. However, a current disadvantage is that SOCS has not yet been integrated with clinical outcomes (e.g., weaning success) nor validated in patient cohorts or against gold-standard modalities.

## 5 Conclusion

In summary, this study presents a pilot feasibility demonstration of an SCBM-based ultrasound speckle-tracking framework for diaphragm deformation analysis. The proposed SOCS method enabled estimation of inter-frame and cumulative displacement as well as approximate global longitudinal strain. In this small cohort of healthy beagles, mean/median displacement summaries across ROI points were comparable between sides, whereas global strain differed between imaging sections/sides. These findings suggest that diaphragm strain estimates may be sensitive to imaging-plane selection. Because this was a small feasibility study without external validation, further work is needed in larger datasets, pathological models, and reference-standard comparisons.

## Data Availability

The raw data supporting the conclusions of this article will be made available by the authors, without undue reservation.
